# Lack of evidence for transcription-to-RNA maturation lags across G1-to-G2-phase boundary in fibroblasts

**DOI:** 10.26508/lsa.202603670

**Published:** 2026-07-22

**Authors:** Melanie Gucwa, Kohta Ikegami

**Affiliations:** 1 Division of Molecular Cardiovascular Biology, Cincinnati Children’s Hospital Medical Center , Cincinnati, OH, USA; 2 Department of Pediatrics, University of Cincinnati College of Medicine, Cincinnati, OH, USA

## Abstract

Cell-cycle dynamics of gene expression are fundamental to life, yet their mechanistic basis remains unclear. A prevailing model, derived from cancer cells, posits that RNAs transcribed in one cell-cycle phase peak in abundance in subsequent phases, suggesting temporal segregation of transcriptional regulation and RNA maturation during the cell cycle. However, whether such extensive transcription-to-maturation lags occur in normal cells remains unclear. Here, we report a lack of evidence for systematic transcription-to-maturation lags extending across the G1-to-G2-phase boundary in normal fibroblasts. Using FUCCI cell-cycle reporters coupled with RNA-seq and GRO-seq, we defined the cell-cycle dynamics of nascent transcription and mature RNA abundance in BJ-5ta human fibroblasts. Transcription and mature RNA accumulation were strongly temporally concordant. Genes with peak transcription in G1, early S, or late S/G2/M showed peak mature RNA abundance in the corresponding phases, without extensive lags. Minor lags within the G1 phase were detected, confirming the sensitivity of our approach. Thus, our data do not support the generality of the prevailing model of extensive transcription-to-accumulation lags in normal human cells.

## Abstract (precis)

In human fibroblasts, transcription and RNA accumulation are temporally concordant across the cell cycle, challenging the model of extensive transcription-to-maturation lags during the cell cycle.

## Introduction

Precise temporal control of gene expression during the cell cycle is essential for cellular homeostasis. Disruption of this regulation can drive uncontrolled proliferation and tumorigenesis (1). More than 1,000 human genes show cell-cycle phase–specific accumulation of transcripts (2, 3, 4, 5, 6, 7). However, the transcriptional mechanisms that generate these dynamics remain controversial. One prevailing model proposes an extensive transcription-to-maturation lag, in which the peak of transcriptional activity in one cell-cycle phase is followed by peak RNA abundance in subsequent phases (8). For example, transcripts produced in early G1 accumulate in the S phase, those produced in the G1/early S phase accumulate in the M phase, and those produced in the M phase accumulate in G0/G1 (8). This model contrasts with the classical examples of cell-cycle gene regulation, in which transcription and transcript accumulation occur within the same cell-cycle phase. For example, E2F1-target genes are transcribed in late G1 and function for G1-to-S progression (9). The proposed systematic lags are unlikely to arise solely from transcription elongation, because transcription of average-sized genes (up to 30 min (10, 11, 12)) is substantially shorter than a typical cell-cycle phase. Instead, this model implies that regulation of transcription and regulation of RNA maturation are temporally segregated during the cell cycle. However, whether this prevailing model, derived from MCF-7 cancer cells arrested at specific cell-cycle phases (8), applies to cycling normal cells remains unclear.

One reason the temporal relationship between transcription and transcript accumulation remains unresolved is that few studies have measured nascent and mature RNAs simultaneously during the cell cycle. A recent single-cell study in HEK293T cells measured nascent and mature RNAs and reported only a subtle lag along a pseudotime scale that did not appear to extend across inferred cell-cycle phases (3). One challenge in studying cell cycle–specific gene expression is that perturbations used to arrest cells at specific cell-cycle phases, such as thymidine block, serum starvation, and nocodazole treatment, can affect gene expression independent of cell-cycle phases *per se* (13, 14, 15). The FUCCI cell-cycle reporter system allows isolation of cells at specific cell-cycle phases without perturbation (16). Its recent version, FUCCI4, is a multi-transgene system that produces fluorescently labeled Cdt1, Geminin, and SLBP miniproteins, which accumulate in specific cell-cycle phases owing to cell cycle–specific degradation (17).

Here, we use FUCCI4 reporters to isolate specific cell-cycle populations of human fibroblasts and coprofile nascent and mature RNAs using GRO-seq and RNA-seq. We find no evidence for a systematic lag between transcription and RNA maturation that extends across the G1-to-G2-phase boundary.

## Results

### FUCCI4 cell-cycle reporters in BJ-5ta human fibroblasts

**Figure 1.**
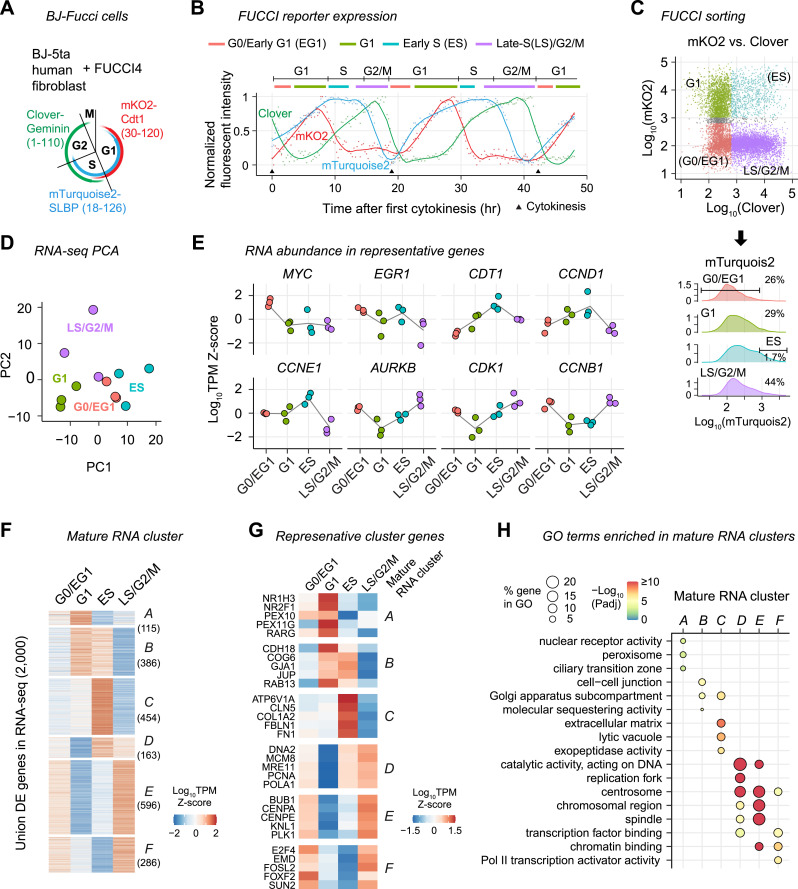
Cell-cycle dynamics of mature RNA in human fibroblasts. **(A)** BJ-Fucci fibroblasts express the FUCCI4 cell-cycle reporters mKO2-Cdt1^aa30-120^, mTurquoise2-SLBP^aa18-126^, Clover-Geminin^aa1-110^, and mMaroon1-H1. **(B)** Nuclear FUCCI4 reporter signals in a single BJ-Fucci cell (0–19 h), daughter cell (19–43 h), and granddaughter cell (43 h onward), measured by time-lapse imaging (48-h duration, 15-min interval). Circle, min–max normalized log_10_ intensity. Line, Loess regression. Top, empirical cell-cycle phase annotations. **(C)** FACS profile of asynchronous BJ-Fucci cells. Cell populations indicated in the Clover/mKO2 plot (top) are shown in the mTurquoise2 histogram (bottom). Percentage, fraction of each population within total sorted cells. Sorted populations are mKO2^–^Clover^–^mTurquoise2^–^ (G0/early G1; G0/EG1), mKO2^+^Clover^–^ (G1), mKO2^+^Clover^+^mTurquoise2^+^ (early S; ES), and mKO2^–^Clover^+^ (late S/G2/M; LS/G2/M). **(D)** Principal component analysis (PCA) of RNA-seq signals in biological replicates (N = 3). **(E)** RNA-seq signals of select genes with known cell cycle–dependent expression. **(F)** K-means clustering of the union of differentially abundant genes based on RNA-seq signal dynamics. Color, gene-level z-score of log_10_ RNA-seq exon TPM (transcripts per million). **(G)** Mature RNA abundance (RNA-seq signal) dynamics of five representative genes from each mature RNA cluster. **(H)** Molecular Function and Cellular Component GO terms enriched in mature RNA clusters. Top 3 enriched terms per cluster are shown. Color, enrichment within each cluster.

**Figure S1.**
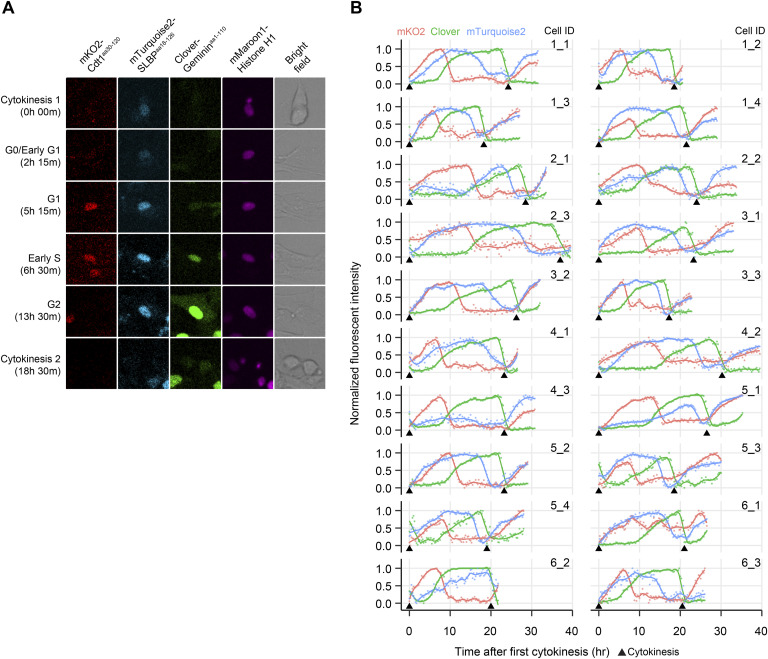
Time-lapse imaging of BJ-Fucci cells. **(A)** Representative BJ-Fucci cell in time-lapse confocal imaging (48-h duration, 15-min interval). Fluorescence signal of mKO2, mTurquoise2, Clover, and mMaroon1 and bright-field image for a single fibroblast at the indicated cell-cycle stage. **(B)** Nuclear fluorescence signals in individual BJ-Fucci cells. Each plot shows one cell cycle and a part of the following cycle of the daughter cell. Circle, min–max normalized log_10_ intensity. The line represents Loess regression. The arrowhead indicates cytokinesis of the daughter cells.

To investigate cell-cycle dynamics of transcription and transcript accumulation in cycling, nontransformed cells, we introduced the FUCCI4 fluorescent cell-cycle reporters (17) into the hTERT-immortalized human fibroblast cell line BJ-5ta (BJ-Fucci) (Fig 1A). The FUCCI4 system consists of four components: Cdt1^aa30-120^ fused to mKusabira-Orange2 (mKO2), which accumulates in G1; SLBP^aa18-126^ fused to mTurquoise2, which accumulates in G1 and S; Geminin^aa1-110^ fused to Clover, which accumulates in S and G2; and histone H1 fused to mMaroon1, which visualizes chromatin (17). We examined FUCCI marker expression using time-lapse imaging (Figs 1B and S1A and B, and Table S1). During early G1 (∼2 h after cytokinesis), mKO2, mTurquoise2, and Clover signals were low or nearly undetectable. This was followed by a peak in mKO2 expression, indicating the G1 phase. mTurquoise2 then reached peak expression as mKO2 began to decline and Clover began to increase, indicating the early S phase. This was followed by a peak in Clover expression, indicating G2/M, followed by cell division and a rapid decline in the Clover signal (Fig S1B and Video 1). These observations validated the expected temporal expression of FUCCI4 markers and established a system for isolating specific cell-cycle phases without cell-cycle arrest.

Table S1. Fucci4 marker signals in time-lapse imaging.

Video 1 Time-lapse imaging of BJ-Fucci cells. Download video

### Mature RNA dynamics during the cell cycle

**Figure S2.**
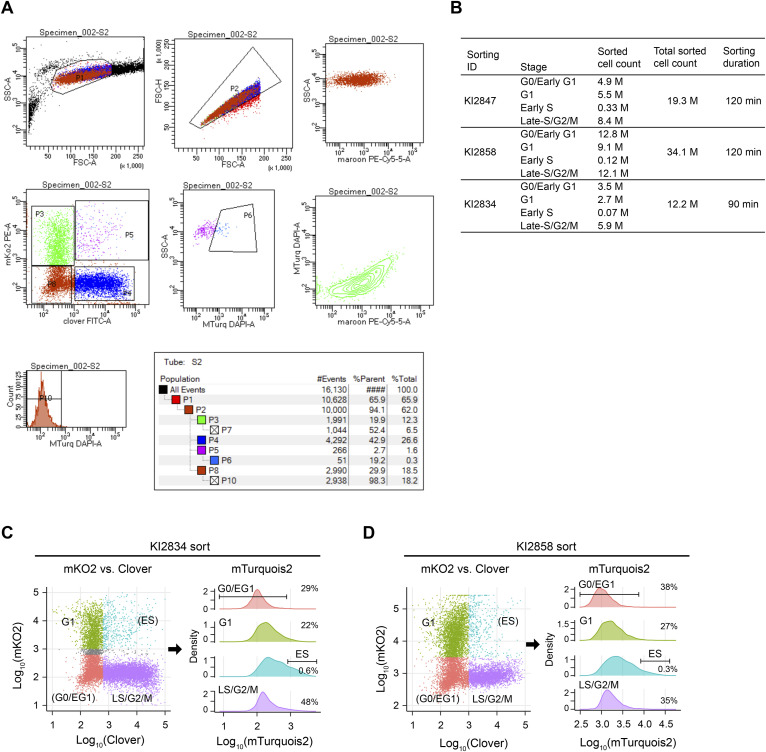
FACS of BJ-Fucci cells. **(A)** Gating strategy for sorting BJ-Fucci cells. P1, cell selection; P2, singlet cell selection; P3, G1; P4, L2/G2/M; P5, parental population of P6; P8, parental population of P10; P6, early S; P10, G0/EG1. **(B)** Summary of sorting experiments. **(C, D)** FACS profile of asynchronous BJ-Fucci cells. Cell populations indicated in the Clover/mKO2 plot (left) are shown in the mTurquoise2 histogram (right). Percentage, fraction of each population within total sorted cells. Sorted populations are mKO2^–^Clover^–^mTurquoise2^–^ (early G1; EG1), mKO2^+^Clover^–^ (G1), mKO2^+^Clover^+^mTurquoise2^+^ (early S; ES), and mKO2^–^Clover^+^ (late S/G2/M; LS/G2/M). Two biological replicates (A, B) of FACS experiments are shown. Another replicate is shown in Fig 1.

**Figure S3.**
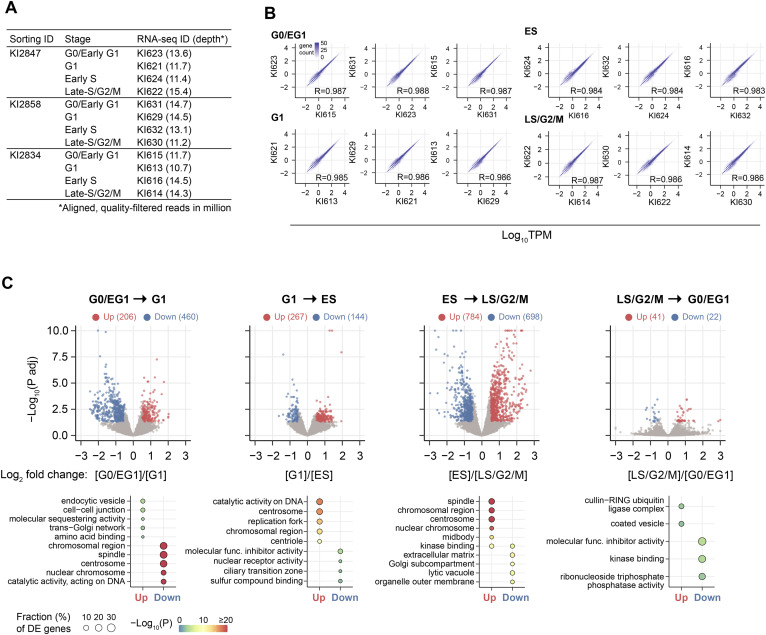
RNA-seq analysis of cell cycle–sorted BJ-Fucci cells. **(A)** RNA-seq datasets generated in this study. **(B)** Pairwise comparison of RNA-seq signals (log_10_TPM) between biological replicates. R, Pearson’s correlation coefficient. **(C)** Differentially abundant genes between neighboring cell-cycle phases. Top, volcano plots showing log_2_ fold change (x-axis) and DESeq2 adjusted *P*-value (y-axis) for all genes. Color, differentially abundant genes. Bottom, five most enriched GO terms among up-regulated and down-regulated genes.

To determine the dynamics of mature RNA abundance during the cell cycle, we sorted BJ-Fucci cells into four phases using fluorescence-activated cell sorting (FACS): G0/early G1 (mKO2^–^Clover^–^mTurquoise2^–^), G1 (mKO2^+^Clover^–^), early S (mKO2^+^Clover^+^mTurquoise2^+^), and late S/G2/M (mKO2^–^Clover^+^) (Figs 1C and S2A–D, and Table S2). We then performed RNA-seq on poly(A)-selected RNAs from each population and quantified mature RNA abundance using read coverage over exons (Fig S3A). We confirmed high consistency between biological replicates and observed separation of cell-cycle phases by principal component analysis (PCA) (Figs 1D and S3B). RNA-seq data recapitulated known cell-cycle gene expression dynamics, including early G1 expression of *MYC* (18) and *EGR1* (19), G1-to-early-S elevation of *CDT1* and *CCND1* (20, 21), peak early S expression of *CCNE1* (20), and late S/G2/M expression of *AURKB*, *CCNB1*, and *CDK1* (20, 22) (Fig 1E). We next identified genes whose mature RNA abundance differed significantly between neighboring cell-cycle phases. We identified 666 differentially abundant genes between G0/early G1 and G1, 411 between G1 and early S, 1,482 between early S and late S/G2/M, and 63 between late S/G2/M and G0/early G1, yielding a total of 2,000 unique genes (Fig S3C). We then clustered these 2,000 genes based on their mature RNA abundance dynamics into six clusters (Clusters *A*–*F*) (Fig 1F and Table S3). Cluster *A* (115 genes) showed peak abundance in G1 and was enriched for Gene Ontology (GO) terms related to nuclear receptors (e.g., *RARG*) and peroxisome components (e.g., *PEX10*) (Fig 1G and H). Clusters *B* (386 genes) and *C* (454 genes) showed peak abundance in G1 and early S (Cluster *B*) or early S (Cluster *C*) and were enriched for fibroblast function–related genes, including those involved in cell–cell junctions and adhesion (e.g., *GJA1*) and extracellular matrix (e.g., *COL1A2*). Cluster *D* (163 genes) showed peak abundance in early S and late S/G2/M and was enriched for DNA replication–related genes (e.g., *PCNA*). Cluster *E* (596 genes) showed peak abundance in late S/G2/M and was enriched for mitosis-related genes (e.g., *CENPE*, *PLK1*). Cluster *F* (286 genes) also showed a peak in late S/G2/M and was enriched for transcription factors (e.g., *E2F1*) and nuclear envelope proteins (e.g., *SUN2*). Notably, no cluster exhibited a distinct peak at G0/early G1. Together, these results defined the cell-cycle dynamics of mature RNA abundance in normal fibroblasts and provided a framework for investigating their relationship to transcription timing.

Table S2. Fucci4 marker signals in FACS.

Table S3. RNA-seq exon TPM, z-score, differentially abundant genes, and clusters.

### Nascent RNA dynamics in BJ-Fucci cells

**Figure 2.**
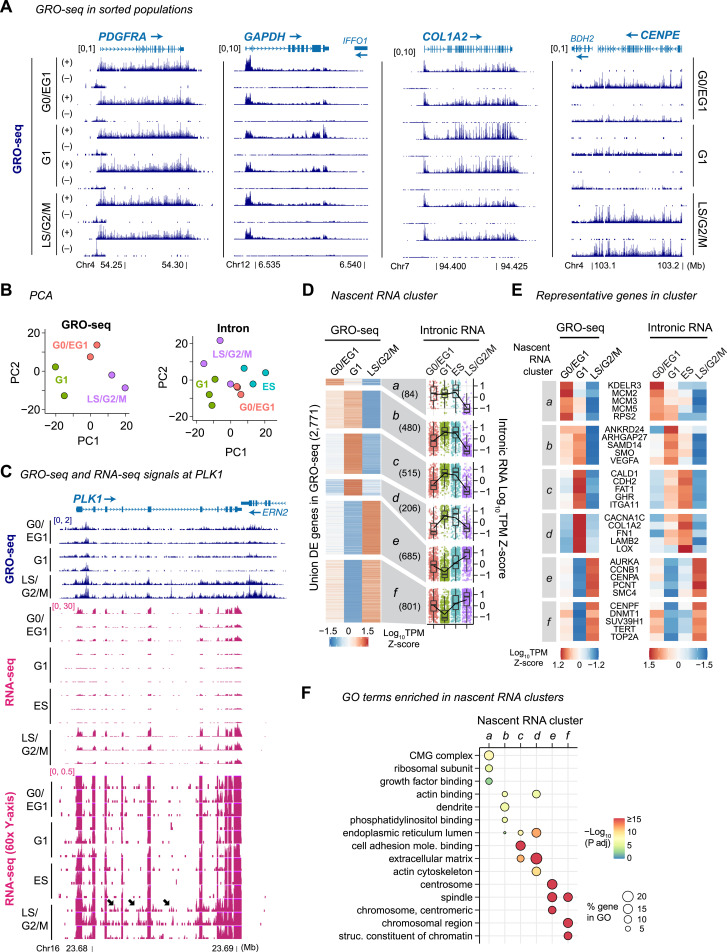
Cell-cycle dynamics of nascent transcription in human fibroblasts. **(A)** GRO-seq signals at select nondynamic (*PDGFRA* and *GAPDH*) and dynamic genes (*COL1A2* and *CENPE*), normalized by sequencing depth. N = 2 per stage. Numbers in brackets indicate y-axis signal range. (+), plus strand. (–), minus strand. **(B)** PCA of GRO-seq and RNA-seq intron signals in biological replicates. **(C)** GRO-seq and RNA-seq read distribution (plus strand) at the *PLK1* locus, normalized by sequencing depth. Bottom RNA-seq y-axis is zoomed 60× to visualize intronic reads. Arrow, representative intron signal. Numbers in brackets indicate y-axis signal range. **(D)** Left, k-means clustering of the union of differentially transcribed genes based on GRO-seq signal dynamics. Color, gene-level z-score of log_10_ GRO-seq TPM. Right, intronic RNA read dynamics for genes in each cluster. Values, gene-level z-score of log_10_ intronic read TPM. Boxplots, interquartile range. Whiskers, farthest data points within 1.5× interquartile range. Line, cluster mean. **(E)** GRO-seq and intronic signal dynamics of five representative genes from each nascent RNA cluster. **(F)** Molecular Function and Cellular Component GO terms enriched in nascent RNA clusters. Top 3 enriched terms per cluster are shown. Color, enrichment within each cluster.

**Figure S4.**
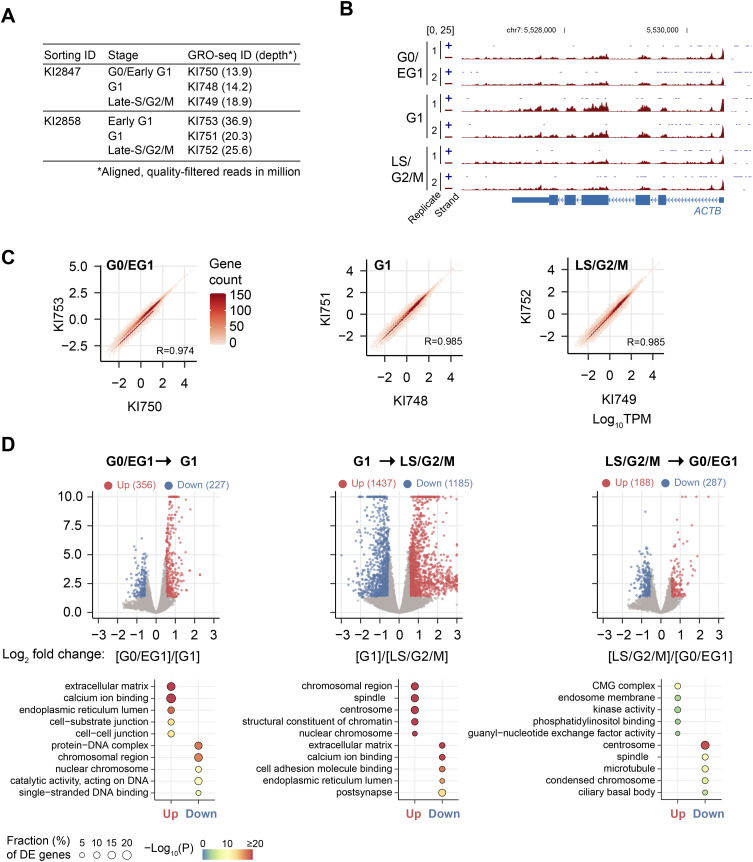
GRO-seq analysis of cell cycle–sorted BJ-Fucci cells. **(A)** GRO-seq datasets generated in this study. **(B)** GRO-seq read density at the *ACTB* locus, normalized to sequencing depth. Plus- and minus-strand signals are shown separately. Numbers in brackets indicate y-axis signal range. **(C)** Pairwise comparison of GRO-seq signals (log_10_TPM) between biological replicates. R, Pearson’s correlation coefficient. **(D)** Differentially transcribed genes between neighboring cell-cycle phases. Top, volcano plots showing log_2_ fold change (x-axis) and DESeq2 adjusted *P*-value (y-axis) for all genes. Color, differentially transcribed genes. Bottom, five most enriched GO terms among up-regulated and down-regulated genes.

To quantify nascent transcripts genome-wide, we performed GRO-seq (23) on nuclei isolated from BJ-Fucci cells sorted at G0/early G1, G1, and late S/G2/M (Figs 2A and S4A, and Table S4). Isolated nuclei were incubated with bromouridine triphosphate for 8 min to label nascent transcripts, which were purified and sequenced for GRO-seq. Of note, we could not obtain GRO-seq data for the early S phase because of insufficient numbers of sorted cells. We confirmed that GRO-seq signals were strand-specific, highly consistent between biological replicates, and originated from both exons and introns, a characteristic feature of nascent transcripts (Figs 2A and S4B and C). GRO-seq signals were largely unchanged across phases at the fibroblast marker *PDGFRA* locus and the housekeeping gene *GAPDH* locus (Fig 2A). In contrast, GRO-seq signals at the collagen *COL1A2* locus were stronger in G1, whereas those at the centromeric protein *CENPE* locus were stronger in late S/G2/M than in other stages (Fig 2A), consistent with the phases in which their mature RNA abundance peaked. PCA confirmed that GRO-seq profiles were distinct across cell-cycle phases (Fig 2B).

Table S4. GRO-seq TPM, z-score, differentially transcribed genes, and clusters.

**Figure S5.**
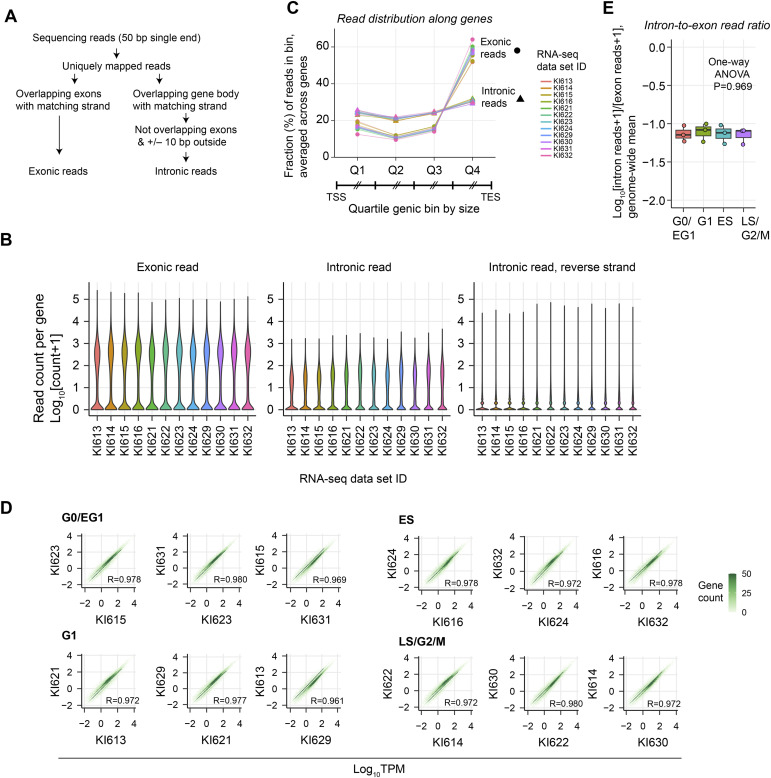
RNA-seq intron signal analysis. **(A)** Flowchart illustrating stratification of exonic and intronic reads from RNA-seq data. **(B)** Total number of exonic and intronic RNA-seq reads mapped to individual genes, shown as distribution of read counts across all genes (y-axis) for each RNA-seq dataset (x-axis). **(C)** Fraction of exonic and intronic RNA-seq reads mapped to indicated gene segments. Values, average fraction across all genes within each RNA-seq dataset. **(D)** Pairwise comparison of intronic RNA-seq signals (log_10_TPM) between biological replicates. R, Pearson’s correlation coefficient. **(E)** Ratio of intronic to exonic RNA-seq reads, representing the relative nascent versus mature RNA abundance. Circle, mean intron/exon ratio across all genes for each biological replicate.

To complement GRO-seq data, we used BJ-Fucci RNA-seq data to quantify nascent transcripts by analyzing reads originating specifically from introns (Figs 2C and S5A, and Table S5) (3, 24, 25). Intronic reads are known to be present in poly(A)-selected RNA-seq because of A-rich sequences in introns and are commonly used to detect nascent transcripts in RNA “velocity” analyses (25, 26). Intronic reads were less abundant than exonic reads, strand-specific, and consistent between replicates, and lacked 3′ enrichment, as expected (Fig S5B–D). The abundance of intronic reads relative to exonic reads was maintained throughout the cell cycle, suggesting the absence of global change in transcriptional activity relative to mature RNA levels during interphase (Fig S5E). PCA showed that the intronic read profiles were also distinct between cell-cycle stages (Fig 2B). For example, intronic reads were abundant at the G2/M kinase *PLK1* locus in late S/G2/M, consistent with high GRO-seq signals in this stage (Fig 2C).

Table S5. RNA-seq intron TPM and z-score.

We used GRO-seq and intron data to define nascent transcript dynamics. We first identified differentially transcribed genes between neighboring cell-cycle phases using GRO-seq data. We identified 583 differentially transcribed genes between G0/early G1 and G1, 2,622 between G1 and late S/G2/M, and 475 between late S/G2/M and G0/early G1, yielding a total of 2,771 unique genes (Fig S4D and Table S4). We clustered these genes into six groups based on GRO-seq data (Clusters *a*–*f*) and then used intronic reads to further characterize transcription dynamics, including the early S phase (Fig 2D). Cluster *a* (84 genes) comprised genes that were transcribed from G0/early G1 to early S and repressed in late S/G2/M (Fig 2D and E). This cluster was enriched for GO terms related to basic cellular functions, including replication origin licensing components of the CMG complex (e.g., *MCM2*) (27) and ribosomal subunits (e.g., *RPS2*) (Fig 2F). Clusters *b* (480 genes), *c* (515 genes), and *d* (206 genes) exhibited high transcription in G1 and early S and were enriched for genes involved in actin binding (e.g., *SAMD14*), adhesion (e.g., *CDH2*), and extracellular matrix (e.g., *COL1A2*, *FN1*). Clusters *e* (685 genes) and *f* (801 genes) showed peak transcription in late S/G2/M and were strongly enriched for mitosis-related genes (e.g., *CENPE*, *CCNB1*). Importantly, the signal dynamics of GRO-seq and intronic reads were concordant at cell-cycle phases where both datasets were available (Fig 2D). Together, using two orthogonal approaches, we defined the cell-cycle dynamics of nascent transcription in BJ-Fucci cells.

### Transcription and mature RNA abundance are highly temporally concordant

**Figure 3.**
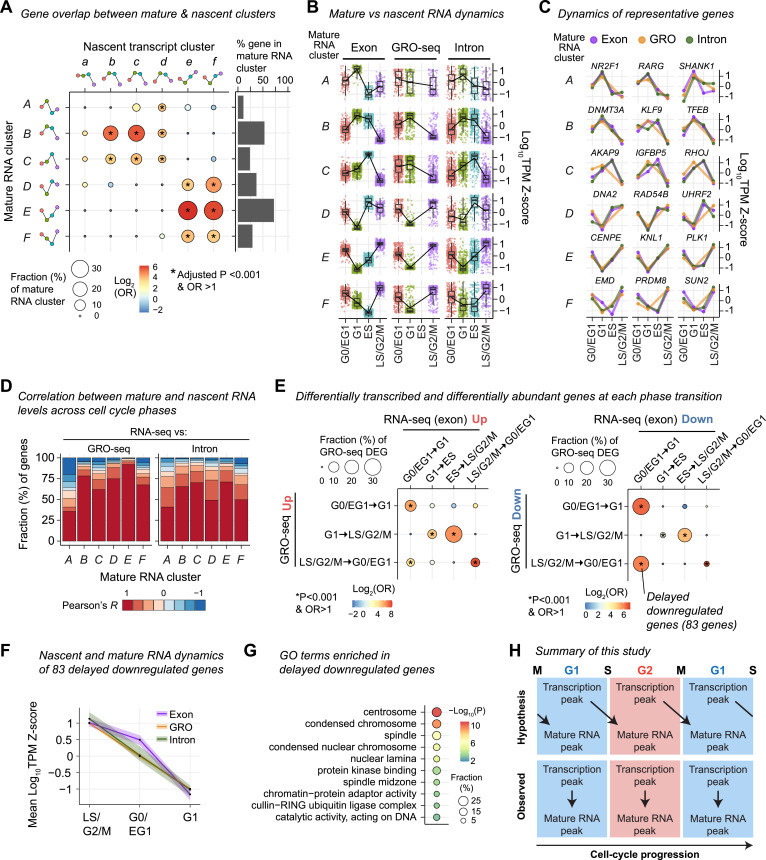
Strong concordance between the cell-cycle dynamics of mature RNA and nascent transcription. **(A)** Left, fraction of genes in mature RNA clusters affiliated with nascent transcriptional clusters. OR, odds ratio. P, Fisher’s exact test *P*-value. Small line plots, schematic representation of cluster dynamics (see Materials and Methods). Right, fraction affiliated with any nascent transcript cluster. **(B, C)** Mature RNA and nascent transcription dynamics for all (B) or representative genes (C) in mature RNA clusters. **(D)** Fraction of genes in mature RNA clusters with indicated Pearson’s correlation coefficients between mature RNA abundance and nascent transcription across cell-cycle phases. **(E)** Fraction of differentially transcribed genes (GRO-seq; y-axis) affiliated with differentially abundant genes (RNA-seq exon; x-axis) at indicated cell-cycle transitions. Left, up-regulated genes. Right, down-regulated genes. Genes down-regulated transcriptionally from LS/G2/M to EG1 but at the mature RNA level during EG1 to G1 are defined as delayed down-regulated genes. **(F)** Mature RNA and nascent transcription dynamics of all delayed down-regulated genes. **(G)** GO terms overrepresented among delayed down-regulated genes. **(H)** Summary. Top: a prevailing hypothesis posits that RNAs transcribed in one cell-cycle phase peak in abundance in subsequent phases. Bottom: contrary to the hypothesis, transcription peaks and mature RNA peaks concur within cell-cycle phases, with no evidence of an extensive lag that extends across the G1/G2 boundary in BJ-5ta human fibroblasts.

We investigated the relationship between cell-cycle dynamics of transcription and mature RNA abundance by assessing the gene overlap between the respective clusters. We observed a highly statistically significant overlap between clusters with similar dynamics. For example, mature RNA clusters with peaks in G1 or early S (Clusters *A*, *B*, *C*) shared genes with transcriptional clusters that peaked in G1 (Clusters *b*, *c, d*) (Fig 3A). Likewise, a significant number of genes overlapped between mature RNA clusters and transcriptional clusters with late S/G2/M peaks (Clusters *D*, *E*, *F*, versus Clusters *e* and *f*). The strongest overlap was observed in late S/G2/M, where 72% of Cluster *E* genes belonged to either Cluster *e* or *f*. Importantly, we did not observe a pattern of overlap that would be expected if a lag extending across phases were present. For example, there was no significant overlap between mature RNA clusters peaking in G1 or early S and transcriptional clusters peaking in late S/G2/M (Fig 3A). Likewise, no significant overlap was observed between mature RNA clusters peaking in late S/G2/M and transcriptional clusters peaking in early G1, G1, or early S. These results suggested that transcription dynamics and mature RNA dynamics were aligned within the same cell-cycle phases and did not exhibit a lag across the G1-to-G2 boundary.

**Figure S6.**
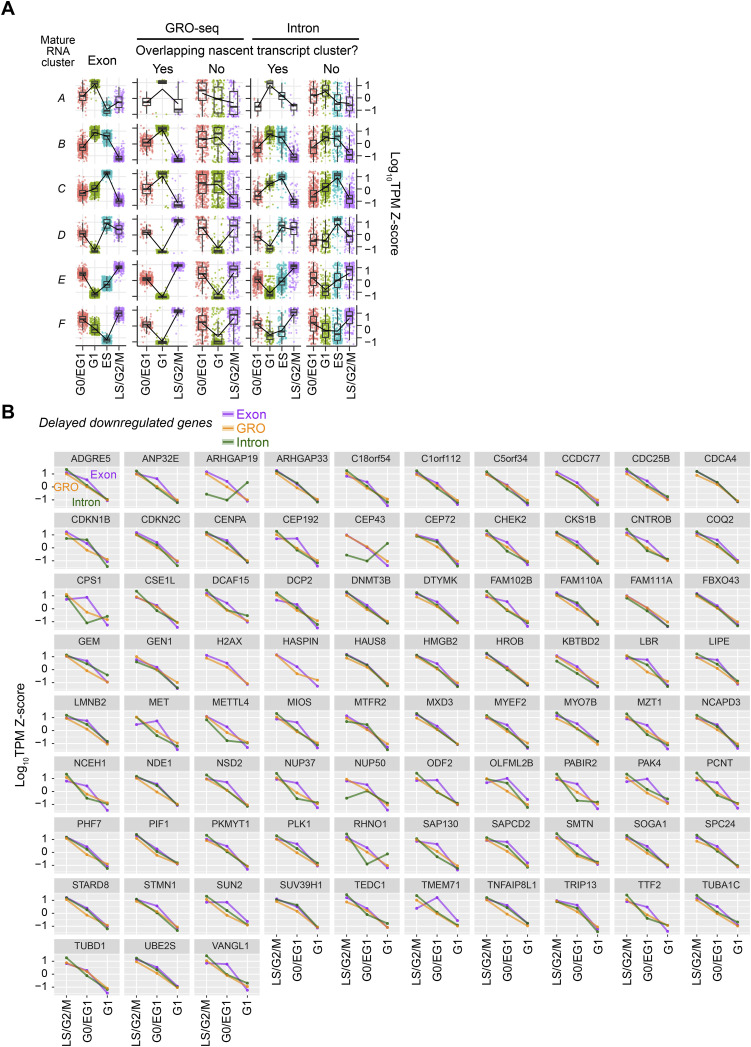
Comparison between mature and nascent RNA dynamics. **(A)** Mature and nascent RNA dynamics of genes stratified by mature RNA clusters and by affiliation with nascent transcriptional clusters. **(B)** Mature and nascent RNA dynamics of the 83 delayed down-regulated genes. H2AX and HASPIN genes lack intronic RNA measurements because of the absence of introns meeting analysis criteria.

We next asked whether the concordance between transcription and mature RNA dynamics was a general phenomenon beyond genes in cluster intersections. We observed that genes in mature RNA clusters exhibited highly concordant nascent transcription dynamics overall (Fig 3B), regardless of their assignment to specific transcriptional clusters (Fig S6A). High concordance between RNA-seq (exon), GRO-seq, and intron read dynamics was also evident at the level of individual genes (Fig 3C). For example, *CENPE*, which was previously reported to exhibit a transcription peak in G1 and a mature RNA peak in the M phase (8), showed highly concordant dynamics, with RNA-seq, GRO-seq, and intron peaks all residing in late S/G2/M. We further computed the correlation between mature and nascent RNA levels across cell-cycle phases for each gene. Over 75% of genes in mature RNA clusters showed a strong positive correlation (Pearson’s *r* > 0.5) between mature and nascent RNA levels throughout the cell cycle (Fig 3D). Thus, the timing of transcription and RNA accumulation generally coincided within the same cell-cycle stages, with no evidence of a systematic and extensive transcription-to-maturation lag extending across the G1-to-G2 boundary in fibroblasts.

### Minor transcription-to-maturation lags within the G1 phase

We next tested whether a transcription-to-maturation lag existed *within* cell-cycle phases. We examined the extent of overlap between differentially transcribed genes (GRO-seq) and differentially abundant genes (RNA-seq exon) at each cell-cycle transition (Fig 3E). We observed that a significant fraction (29%) of genes transcriptionally down-regulated from late S/G2/M to early G1 by GRO-seq were down-regulated at the mature RNA level later, from early G1 to G1 (Fig 3E, right). This lag was further validated by differences in down-regulation timing between intronic and exonic reads in RNA-seq (Figs 3F and S6B). These “delayed” down-regulated genes (83 genes) were strongly enriched for mitosis-related genes (e.g., *PLK1*, *LMNB2*) (Fig 3G), suggesting that these genes were transcriptionally repressed in early G1, whereas their mature transcripts persisted until later in G1. A similar, though weaker, within-G1 lag was observed for a subset (5%) of genes transcriptionally up-regulated from late S/G2/M to early G1, which showed up-regulation at the mature RNA level later, from early G1 to G1 (Fig 3E, left). Taken together, the cell-cycle dynamics of transcription and mature RNA accumulation were highly concordant, with only a minor lag detected within G1 in BJ-Fucci human fibroblasts (Fig 3H).

## Discussion

Our analysis revealed strong consistency between the cell-cycle dynamics of nascent transcription and mature RNA abundance in normal human fibroblasts using sorted cell-cycle populations (Fig 3H). We did not find evidence for a widespread transcription-to-maturation lag extending across the G1-to-G2 boundary. This conclusion differs from observations of a pervasive lag extending across the G1-to-G2 boundary reported in the cancer cell line MCF-7 (8). In contrast, our finding is consistent with a recent single-cell transcriptome study in HEK293T cells, which measured nascent and mature RNAs using exonic and intronic reads and reported only a minor lag along pseudotime (3). Although additional cell types need to be examined, our observations question the generality of the previously proposed extensive cross-phase transcription-to-maturation lags.

There are several possible biological and technical explanations for the discrepancy between our observations and those reported in MCF-7 cells (8). First, the discrepancy may relate to differences in cell-cycle progression time, which is faster in MCF-7 cells (8) than in BJ-5ta cells used in this study. For a given RNA half-life, a transcription-to-maturation lag would occupy a larger fraction of the cell cycle in rapidly cycling cells. However, this does not fully explain the absence of cross-phase lags observed in HEK293T cells, which also cycle rapidly (3). Other possible biological contributors include dysregulation of transcription and/or RNA turnover in certain cancer cells, cell-type differences, and differences in transformation state. Technical factors may also contribute. Although the MCF-7 study examined cells arrested at defined cell-cycle phases (8), both our study and the HEK293T single-cell study (3) used unperturbed, normally cycling cells. Another difference is that the MCF-7 study did not include G2-phase cells, whereas the present study did. In the MCF7 study, many genes with RNA-seq peaks in the M phase, including the centromeric protein gene *CENPE*, show transcription peaks in G1 (8). However, this may reflect exclusion of G2 cells, because we observed that many mitosis-related genes, including *CENPE*, exhibited strong transcription peaks in late S/G2/M cells.

Unlike the *cross-phase* lags that we did not detect, our study identified a minor *within-phase* lag in G1. Specifically, we found that most of the mitosis-related genes were transcriptionally repressed in early G1, whereas their mature RNAs declined later in G1. This observation is consistent with a previous study showing that most mRNAs of mitosis-related genes are degraded during G1, not immediately after mitotic exit (28). Detection of this minor lag supports the sensitivity of our approach to identify larger systematic lags, if present. We predict that similar minor within-phase lags occur throughout the cell cycle, as observed in a previous single-cell study in HEK293T cells (3), because RNA processing and degradation inherently require time.

This study has several limitations. First, our analysis normalizes data such that global transcriptional activity and total mature RNA abundance remain constant across the cell cycle. In reality, nascent transcription may be globally higher in G2 than in other phases because of genome duplication (29). However, even if such global changes occur, our central conclusion would remain unaffected because the abundance of mature RNA relative to that of nascent RNA was globally maintained across the analyzed phases based on intron-to-exon ratio analysis (Fig S5E). Second, cell-cycle phases defined by the FUCCI system may not precisely correspond to classical phase definitions based on DNA content. Although this difference may affect biological interpretation of cell-cycle dynamics of individual genes, it does not alter our central conclusion because the temporal relationships among FUCCI-defined populations are fixed. Third, sorted populations were heterogeneous with respect to cell-cycle position. This heterogeneity could arise from inclusion of distinct cell-cycle phases within the G0/early G1 and late S/G2/M populations, as well as variation within phases, such as G1 and early S. In addition, minor contamination from other phases may occur because of fluctuations in FUCCI marker expression (Figs 1B and S1B). This heterogeneity and potential contamination could blur cell-cycle dynamics and potentially reduce sensitivity for detecting certain transcription-to-maturation lags. Nevertheless, the identification of more than 2,000 genes with mature and nascent RNA dynamics, many showing biologically relevant patterns, supports the sensitivity of our methodology.

In conclusion, our data from BJ-5ta human fibroblasts do not support the prevailing model of transcription-to-maturation lags extending across the G1-to-G2 boundary. Further investigation of the relationship between transcription and RNA maturation during the cell cycle is warranted.

## Materials and Methods

### BJ-Fucci cell line

The hTERT-immortalized human dermal fibroblast cell line BJ-5ta (source: foreskin of male neonate) was purchased from the ATCC (catalog # CRL-4001; ATCC). BJ-5ta retained normal fibroblast growth phenotypes and did not exhibit transformed phenotypes, as described in the original publication (30). To introduce FUCCI4 fluorescent markers, we produced lentiviruses carrying Clover-Geminin^aa1-110^-IRES-mKO2-Cdt1^aa30-120^ (gift from Michael Lin; Addgene ID83841; RRID:Addgene_83841) and lentiviruses carrying mTurquoise2-SLBP^aa18-126^-IRES-H1-mMaroon1 (gift from Michael Lin; Addgene ID83842; RRID:Addgene_83842). A detailed procedure for lentivirus production using HEK293FT cells is described in our previous publication (31). BJ-5ta cells were cotransduced with the two lentivirus supernatants at a 1:60 dilution in the presence of 6.7 μg/ml polybrene. The transduced cells were selected for their simultaneous expression of Clover and mMaroon1 and then for their simultaneous expression of mKO2 and mMaroon1 using FACS. The resultant double-selected cells are “BJ-Fucci” cells (cell ID cc1357-2ss). Independently, we transduced BJ-5ta cells with the two vectors individually (cell ID cc1376-1 for mKO2/Clover and cc1376-2 for mTurquoise2/mMaroon1) or with mKO2-SLBP^aa18-126^ (gift from Michael Lin; Addgene ID 83914; RRID:Addgene_83914; cell ID cc1376-3) or with Clover-Geminin^aa1-110^ (gift from Michael Lin; Addgene ID 83915; RRID:Addgene_83915; cell ID cc1376-4), to generate references for signal compensation in flow cytometry. BJ-5ta and all its derivatives, including BJ-Fucci cells, were cultured in high-glucose DMEM (11965-092; Gibco) containing 9% fetal bovine serum, 90 U/ml penicillin, and 90 μg/ml streptomycin at 37°C under 5% CO_2_.

### Time-lapse confocal microscopy

BJ-Fucci cells in a glass-bottom dish (MatTek P35G-1.5-20-C) were placed in a microscope stage-top incubator set to 37°C and 5% CO_2_ (Tokai Hit) and imaged in 6 areas in the dish every 15 min for 48 h using a Nikon AXR inverted confocal laser scanning microscope with a 20× objective. For each frame for each image area, mTurquoise2, Clover, mKO2, mMaroon1, and bright-field images were obtained. The following excitation (ex) lasers and emission (em) tunable filter settings were used: mTurquoise2, ex 445 nm, em 464–497 nm; Clover, ex 488 nm, em 505–547 nm; mKO2, ex 561 nm, em 571–591 nm; mMaroon1, ex 594 nm, em 609–712 nm. To plot the cell-cycle dynamics of fluorescence signal intensities per cell, we first identified cells that exhibited two cytokinesis events in the imaging area within the imaging duration and obtained mean nuclear fluorescence intensities at each frame (Table S1). We then normalized the log_10_-scaled intensities for each cell such that the minimum intensity was set to 0 and the maximum intensity was set to 1 during the cell cycle, to use in plots.

### Fluorescence-activated cell sorting

Before sorting, fluorescence compensation was set up using BJ-5ta cell populations expressing mKO2-SLBP alone, Clover-Geminin alone, Clover-Geminin-IRES-mKO2-Cdt1 alone, or mTurquoise2-SLBP-IRES-H1-mMaroon1 alone. Approximately 20 h before sorting, BJ-Fucci cells that reached confluency were passaged to 8- to 20 15-cm culture dishes at a density of 2.5 million cells per dish. This passaging schedule increased the proportion of G2/M and early G1 cells, at the expense of the S-phase proportion at the time of sorting. Approximately 30–60 million cells were dissociated from the dishes with TrypLE (Thermo Fisher Scientific), filtered through a 40-μm cell strainer, and resuspended in the complete culture medium for sorting. Cells were sorted into the culture medium using BD FACSAria II (sorting experiment ID: KI2834 and KI2847) or BD FACSAria IIu (KI2858) sorters. The following populations were sorted: mKO2^–^Clover^–^mTurquoise2^–^ (G0/early G1), mKO2^+^Clover^–^ (G1), mKO2^+^Clover^+^mTurquoise2^+^ (early S), and mKO2^–^Clover^+^ (late S/G2/M). The following ex lasers and em filter settings were used: mTurquoise2, ex 405 nm, em 450/50 nm; Clover, ex 488 nm, em 530/30 nm; mKO2, ex 561 nm, em 582/15 nm; and mMaroon1, ex 561 nm, em 670/30 nm (FACSAria II) or em 670/14 (FACSAria IIu). Sorting was performed for 90 min (KI2834) or 120 min (KI2847 and KI2858), during which we collected as many cells as possible (see Fig S2B for sorted cell count). Presorted cells were kept at room temperature, and postsorted cells were moved to ice approximately every 15 min during sorting. After sorting, cells were stored with TRIzol LS (Thermo Fisher Scientific) at –80°C for RNA-seq or directly proceeded to nuclear isolation described in the GRO-seq section. Fluorescence signals of cells immediately before sorting are listed in Table S2.

### RNA-seq

Total RNAs were purified by TRIzol LS (10296028; Invitrogen) and treated with DNase I (AM2238; Invitrogen Turbo DNase). RNAs were isolated and fragmented using NEBNext Poly(A) mRNA Magnetic Isolation Module (E7490; New England Biolabs). cDNA libraries were prepared using NEBNext Ultra II Directional RNA Library Prep Kit for Illumina (E7660; NEB). Libraries were processed on the Illumina HiSeq 2500 for single-end 50-nt sequencing.

### GRO-seq

Nuclei were isolated by incubating cells in hypotonic NP-40 lysis buffer (10 mM NaCl, 3 mM MgCl2, 0.5% NP-40, 10 mM Tris, pH 7.5) supplemented with RNase inhibitor on ice. The isolated nuclei were stored in nuclear storage buffer (50 mM Tris, pH 8.0, 0.1 mM EDTA, 5 mM MgCl2, 40% glycerol, RNase inhibitor) at –80°C. For nuclear run-on experiments, the nuclear suspension was mixed with an equal volume of 2× NRO buffer (10 mM Tris, pH 8.0, 5 mM MgCl2, 1 mM DTT, 300 mM KCl, 0.5 mM ATP, 0.5 mM GTP, 0.5 mM bromouridine triphosphate, 2 μM CTP) and incubated for 4 min at 30°C, and then supplemented with 0.5% sarkosyl and incubated for 4 min at 30°C (total of 8 min). RNAs were purified from the reaction by TRIzol LS (10296028; Invitrogen) followed by isopropanol precipitation. RNAs were treated with Turbo DNase (AM18907; Ambion) and fragmented by fragmentation buffer (AM8740; Ambion). BrU-incorporated RNA fragments were immunoprecipitated with mouse monoclonal anti-BrdU antibody 3D4 (555627 Lot 7033666; BD Biosciences) and used to construct DNA sequencing libraries using NEBNext Ultra II Directional RNA Library Prep Kit (E7660; New England Biolabs). DNA libraries were processed on an Illumina NextSeq sequencer for paired-end 42-nt sequencing.

### Genome and gene annotation

We used GRCh38 as the human reference genome and the GENCODE v38 basic gene annotation (60,649 genes and 111,832 transcripts) as the gene reference. For gene-level analyses, we selected a single transcript for each gene by choosing the transcript with the largest total exon length and used its transcript start and end coordinates to represent that gene. The gene_type field in the gene annotation was used to subset genes by type.

### RNA-seq read alignment and counting

RNA-seq reads were aligned to the GRCh38 human reference genome with the GENCODE v38 basic gene annotation (60,649 genes) (32) using STAR 2.7.9 (33) with the default alignment parameters except using “--clip3pAdapterSeq AGA​TCG​GAA​GAG​CAC​ACG​TCT​GAA​CTC​CAG​TCA.” Reads overlapping exons of the GENCODE v38 annotation were counted using the “--quantMode GeneCounts –sjdbGTFfeatureExon exon” options within the STAR alignment run. The bedGraph files of aligned reads normalized by mapped reads were generated using the “--outWigType bedGraph --outWigNorm RPM” options within the STAR process and converted to BigWig files using the bedGraphToBigWig function in UCSC tools (34).

### GRO-seq read alignment and counting

GRO-seq read pairs with a maximum fragment length of 2,000 bp were aligned to the GRCh38 human reference genome using Bowtie2 with the parameters “-X 2000 --no-mixed --no-discordant.” Reads with a MAPQ score greater than 20 were used for downstream analyses. Because the 5′ end of the paired-end fragment approximates the initial location of RNA polymerase II in the run-on experiment, we counted the 5′ end (1 base) of paired-end fragments overlapping transcript coordinates in the GENCODE v38 basic gene annotation (111,832 transcripts) and selected a single transcript per gene with the largest total exon length (see *Genome and gene annotation*) for downstream analyses. To generate genomic density profiles for visualization, we extended ±10 bp from the 5′ end of each fragment and counted overlapping fragments at every base in the genome, and then normalized the counts by the total number of aligned fragments.

### Intron read analysis

To count RNA-seq reads overlapping specifically introns, we made a BED file for all introns by subtracting exon coordinates (from any gene) from the coordinates of the 60,649 genes, restricting the subtraction to cases in which exon orientation matched gene orientation, using BEDTools *subtract* (35). Intron coordinates were further trimmed 10 bp from either side to avoid counting exon-spanning reads misaligned to intronic segments, as recommended in a previous publication (25). These processes resulted in 37,725 genes with countable introns. Using Subread *featureCounts* (36), we counted reads overlapping intronic regions for each gene in the same orientation, as well as those overlapping in the opposite orientation, as a control.

### Batch correction, transcripts per million, and z-score

We performed the following processing steps for RNA-seq exon read counts, RNA-seq intron read counts, and GRO-seq fragment counts. (1) Batch correction: raw read or fragment counts were corrected for sorting batch using ComBat-seq (37). (2) Transcripts per million: the corrected counts were used to compute transcripts per million (TPM), defined as: TPM = 10^6^ x RPK/sum(RPK), where RPK is [1+corrected read count]/[target length in kb], and sum(RPK) is the sum of RPK values for all genes. For target lengths, we used the sum of exon lengths for RNA-seq exon analysis, the sum of intron lengths for RNA-seq intron analysis, and gene lengths for GRO-seq. (3) Z-score: we calculated log_10_TPM for each biological replicate, then computed the mean across replicates within each cell-cycle stage, and finally calculated gene-wise z-scores of these means. The batch-corrected counts, TPM, and z-scores are listed in Table S3 (RNA-seq exon), Table S4 (GRO-seq), and Table S5 (RNA-seq intron).

### Principal component analysis

Genes with no read count in any replicate were removed, and then sample-wise mean-centered log_10_TPM values were used as input to the *prcomp* function in R.

### Differentially abundant and differentially transcribed genes

Corrected counts for RNA-seq exon reads or GRO-seq fragments were analyzed using the DESeq2 program (38) to identify differentially expressed genes. Genes with adjusted *P* <0.05 and an absolute log_2_ fold change >0.5 were considered differentially abundant (RNA-seq) or differentially transcribed (GRO-seq). The following stages were compared: between G0/early G1 and G1, between G1 and early S, between early S and late S/G2/M, and between late S/G2/M and G0/early G1 in RNA-seq; and between G0/early G1 and G1, between G1 and late S/G2/M, and between late S/G2/M and G0/early G1 in GRO-seq. The identified genes are indicated in Table S3 (RNA-seq) or Table S4 (GRO-seq).

### Gene clustering

We identified groups of genes with similar cell-cycle dynamics within the union of differentially abundant genes (2,000 genes) or the union of differentially transcribed genes (2,771 genes). We applied k-means clustering with k = 6 to the z-scores of log_10_TPM for each union set. The mean z-score across genes within each cluster was used to generate schematic representations of cluster dynamics. The clusters are indicated in Table S3 (RNA-seq) or Table S4 (GRO-seq).

### GO analysis

For GO enrichment analysis, genes annotated as “protein_coding” in the GENCODE v38 gene_type field were analyzed in Metascape (39) using the default settings, except that we selected GO Molecular Functions and GO Cellular Components as the search databases.

### Gene overlap analysis

The statistical significance of gene overlap between two gene sets was assessed using Fisher’s exact test. *P*-values were adjusted for multiple comparisons using the Benjamini–Hochberg procedure.

### Correlation of z-scores

We used z-scores of log_10_TPM for correlation analysis. For each gene, z-scores across cell-cycle stages served as the shared data points. Pearson’s correlation coefficients were computed either between RNA-seq exonic z-scores and GRO-seq z-scores, or between RNA-seq exonic z-scores and RNA-seq intronic z-scores. In the RNA-seq versus GRO-seq comparisons, only the shared stages (i.e., excluding the early S data points from RNA-seq) were used.

### RNA-seq read distribution along genes

We first extracted RNA-seq reads overlapping exons and reads overlapping introns trimmed by 10 bp at each end. We then defined genomic coordinates for four equally sized bins along each gene. Exonic and intronic reads overlapping each bin were counted using Subread *featureCounts*, and the fraction of reads overlapping each of the bins was computed for each gene.

## Supplementary Material

Reviewer comments

## Data Availability

RNA-seq and GRO-seq data are available in Gene Expression Omnibus under accession ID GSE305617. Computer codes, and unprocessed and processed data used in this study are available in GitHub (https://github.com/kohta-ikegami/FUCCI) and Zenodo (https://zenodo.org/records/17934168). The BJ-Fucci cell line is available upon request to the corresponding author under material transfer agreement.
