# Supplementary material for: Lack of evidence for transcription-to-RNA maturation lags across G1-to-G2-phase boundary in fibroblasts
Source: Life Sci Alliance. 2026 Jul 22;9(10):e202603670. doi: 10.26508/lsa.202603670 (PMC13392360; doi:10.26508/lsa.202603670)
Supplement: Reviewer comments [file LSA-2026-03670_review_history.pdf]

Lack of evidence for transcription-to-RNA maturation lags across G1/G2 phase boundary in fibroblasts

Melanie Gucwa and Kohta Ikegami
DOI: https://doi.org/10.26508/lsa.202603670

Corresponding author(s): Kohta Ikegami, Cincinnati Children's Hospital Medical Center

Review Timeline:

Submission Date:	2026-02-14
Editorial Decision:	2026-04-13
Revision Received:	2026-06-08
Editorial Decision:	2026-07-02
Revision Received:	2026-07-06
Accepted:	2026-07-08

Scientific Editor: Sarita Hebbar

Transaction Report:

(Note: With the exception of the correction of typographical or spelling errors that could be a source of ambiguity, letters and reports are not edited. The original formatting of letters and referee reports may not be reflected in this compilation.)

April 13, 2026

Re: Life Science Alliance manuscript #LSA-2026-03670-T

Kohta Ikegami
Cincinnati Children's Hospital Medical Center
Division of Molecular Cardiovascular Biology

Dear Dr. Ikegami,

Thank you for submitting your manuscript entitled "Lack of evidence for transcription-to-RNA maturation lags extending across cell-cycle phases in fibroblasts" to Life Science Alliance.

Your manuscript was reviewed by three expert reviewers whose comments are appended below. As you will note, all the reviewers appreciated the technical quality of your work. That said, the reviews have highlighted some concerns. We invite you to submit a revised manuscript addressing their following concerns:

- Reviewer 2 notes an insufficient temporal resolution in your approach, limiting that the central claim can be applied across all cell cycle phases. We encourage you to follow the suggestions of this reviewer to address this concern or in the least moderate conclusions accordingly throughout the manuscript text to resolve this particular concern.
- Expansion of discussion on gene-level comparisons between existing publications and this work (Reviewer 1 major point, and Reviewer 2, minor point),
- Accurately and completely describe the methods and results (all the reviewers' minor points and additionally the major point of Reviewer 1, major point Reviewer 3).

When submitting the revision, please include a letter addressing the reviewers' comments point by point. While a rebuttal must respond to all points in some form, additional experiments to resolve these points, other than indicated above, are not required.

I would be happy to discuss the revision in more detail via email or phone/videoconferencing. Please let me know which option you prefer, if any.

To upload the revised version of your manuscript, please log in to your account: https://lsa.msubmit.net/cgi-bin/main.plex

You will be guided to complete the submission of your revised manuscript and to fill in all necessary information. Please get in touch in case you do not know or remember your login name.

The typical timeframe for revisions is three months. Please note that papers are generally considered through only one revision cycle, so strong support from the referees on the revised version is needed for acceptance.

While you are revising your manuscript, please also attend to the below editorial points to help expedite the publication of your manuscript. Please direct any editorial questions to the journal office.

Thank you for this interesting contribution to Life Science Alliance. We hope that the comments below will prove constructive as your work progresses, and we are looking forward to receiving your revised manuscript.

Sincerely,

Sarita Hebbar, PhD
Scientific Editor
Life Science Alliance
http://www.lsajournal.org

A. THESE ITEMS ARE REQUIRED FOR REVISIONS

-- A letter addressing the reviewers' comments point by point.

-- An editable version of the final text (.DOC or .DOCX) is needed for copyediting (no PDFs).

-- High-resolution figure, supplementary figure and video files uploaded as individual files: See our detailed guidelines for

preparing your production-ready images, https://www.life-science-alliance.org/authors

-- Summary blurb (enter in submission system): A short text summarizing in a single sentence the study (max. 200 characters including spaces). This text is used in conjunction with the titles of papers, hence should be informative and complementary to the title and running title. It should describe the context and significance of the findings for a general readership; it should be written in the present tense and refer to the work in the third person. Author names should not be mentioned.

-- By submitting a revision, you attest that you are aware of our payment policies found here: https://www.life-science-alliance.org/copyright-license-fee

B. MANUSCRIPT ORGANIZATION AND FORMATTING:

Full guidelines are available on our Instructions for Authors page, https://www.life-science-alliance.org/authors

We encourage our authors to provide original source data, particularly uncropped/-processed electrophoretic blots and spreadsheets for the main figures of the manuscript. If you would like to add source data, we would welcome one PDF/Excel-file per figure for this information. These files will be linked as supplementary "Source Data" files.

IMPORTANT: It is Life Science Alliance policy that if requested, original data images must be made available. Failure to provide original images upon request will result in unavoidable delays in publication. Please ensure that you have access to all original microscopy and blot data images before submitting your revision.

Reviewer #1 (Comments to the Authors (Required)):

General comments
Gucwa and Ikegami investigate the systematic temporal lag between the peak of nascent transcription and the peak of mature RNA accumulation across cell-cycle phases, and important and still elusive question in cell biology. Using FUCCI4 fluorescent reporters in the hTERT-immortalized in BJ human fibroblasts, the authors sort cells into four populations (early G1, G1, early S, late S/G2/M). Then, nascent transcription is measured by GRO-seq and mature RNA transcription by RNA-seq, using exonic and intronic reads as orthogonal readouts. The central finding of the study is that transcription and abundance of mature RNAs are strongly concordant temporally across the phase of the cell cycle, contradicting extensive lags between transcription and maturation reported in MCF7 cancer cells (Liu et al, 2017). Other findings are:
- Genes transcribed in specific phase accumulate mature RNA in the same phase, as assessed by GRO-seq and RNA-seq.
- Within the G1 phase, a minor lag exists for a subset of mitosis-associated genes (e.g., PLK1, LMNB2): transcripts persist briefly after transcriptional repression at the M-to-G1 transition, inherent to normal turnover dynamics.
- The use of FUCCI4 provides a cleaner framework than chemical arrests used broadly in the field.

The study is technically sound and rigorous, and well executed, with an strategy that is appropriate (GRO-seq + intronic reads). Analysis/correlation statistics are convincing, and the RNA-seq data recapitulates well known gene expression patterns. Experimental system thus seem solid and trustworthy. The study uses clear and concise language, with well-organized and easy to read figures. I appreciated that the authors appropriately acknowledge the need to examine additional cell types and some limitations of the FUCCI system versus DNA content-based definition of phases of the cell cycle.
Overall, the claims are well supported by the data and represent a meaningful contribution to the field, by challenging a prevailing model and stressing the importance of studying the cell cycle in non-transformed cells. I recommend acceptance subject to minor revisions (see below).

Major concerns
I don't have any major concerns for this paper. The following points deserve attention:
- No early S GRO-seq data. The use of intronic read as substitute is acceptable but authors should clearly state in the result section that GRO-seq do not cover early S phase. Also would be helpful to explicitly highlight that GRO-seq and intronic read signal concord at phases where both are available.
- Both explanations for discrepancy with Liu et al data in MCF7 cells are plausible. However, the discussion would benefit from a brief comparison of gene-levels. For example, I was wondering whether genes previously reported to show lag appear among the list of concordant genes?
- Global RNA levels: the authors could discuss how variations in global RNA levels across the cell cycle would impact or bias their analysis.

Minor comments
- A couple figure reference issues are found in the manuscript
- Figure 2C: The gene counts per nascent cluster in text (e.g. cluster a: 88, b: 531) do not match the numbers visible in the figure panels (84, 480, 515, 206, 685, 801).
- Results: "early S phase did yield sufficient cell numbers for GRO-seq" I assume it should be did NOT yield

- Discussion: "A recent single-cell transcriptome study in HEK293T cells also reported only a minor lag" could the authors briefly compare the methodological difference with their study
- I think the paper would be strengthened by a brief validation of GRO-seq signal using a housekeeping gene for example to confirm an comparable efficiency across the phases.

Reviewer #2 (Comments to the Authors (Required)):

General assessment
In their work Gucwa and Ikegami couple an advanced cell cycle reporter with RNA-seq and GRO-seq to study the change of transcriptional landscape during cell cycle progression in human non-transformed cells. This reviewer does not have the technical expertise to comment on the RNA-seq and GRO-seq experiments in detail; therefore, my revision will be limited to the cell cycle aspect and the general relevance of the manuscript.
From a relevance standpoint, Gucwa and Ikegami provide a highly curated transcriptomic dataset that is currently missing for human untransformed cells. Their choice of model system is also highly relevant: they use mesenchymal BJ-5ta cells while most modern studies on the cell cycle focus on epithelial RPE-1 cells.

Major Point
Although I generally consider the manuscript solid and the data of high quality, I have one major critique that I strongly believe the authors should address before publication. The main claim of the work, as it stands, is "Lack of evidence for transcription-to-RNA maturation lags extending across cell-cycle phases in fibroblasts". I consider this statement problematic because the "lack of evidence" could simply be attributed to technical limitations.
In this regard, I note that the temporal resolution of the dataset is limited, particularly in the GRO-seq experiments. In Figure 2, the time points for GRO-seq represent only Early G1, G1, and Late S/G2/M pooled together. This lack of temporal resolution severely hinders the central conclusion of the manuscript. One could claim that transcription peak and mRNA peak fall in the same phase only because there is insufficient distinction between phases. Critically, this limitation extends to the RNA-seq data as well: late S, G2, and M are also pooled into a single fraction on the mature RNA side, meaning the authors lack resolution to detect lags within that window on either the nascent or mature side of the comparison.
It is worth noting that the only cell-cycle transition where the authors have genuinely fine-grained coverage on both the nascent and mature RNA side is around G1, and that is precisely where they detect a minor lag. Rather than merely being consistent with a resolution limitation, this observation is actively predicted by it: if lags are only detectable where temporal resolution is sufficient, the simplest interpretation is that the method is underpowered to detect them elsewhere, not that they are absent.
At the moment, the only solid conclusion is that there is a lack of evidence for transcription-to-RNA maturation lags extending across the G1/S transition. I believe the conclusions would be significantly strengthened if the authors could include more precise timing. This could be achieved in a number of orthogonal ways:
• While it might be hard to separate S and G2 experimentally, it is possible to separate M phase reasonably well (e.g. by mitotic shake-off or phospho-histone H3 staining). This would immediately test whether lags from M-phase transcription to G1 mature RNA are hidden in the current pooled bin.
• The authors could synchronize their cells with thymidine or palbociclib. Although these drugs are known to alter the cell cycle profile, they do offer better temporal resolution and could serve as an orthogonal validation of the existing dataset.

Minor points
• It would be important to show how clean a sample the authors can isolate through cell sorting. I would like to see a PI (propidium iodide) profile of the various sorted populations to assess potential contamination from cells in different cell cycle phases.
• What happens to G0 cells with this FUCCI reporter? Is it not expressed? If so, how do the authors distinguish G0 from early G1 cells? BJ-5ta fibroblasts will have some fraction of quiescent cells, and the FUCCI4 system does not cleanly distinguish G0 from early G1, as both populations would be triple-negative for mKO2/Clover/mTurquoise2. If the early G1 bin is contaminated with G0 cells, this could blur the transcriptional signature of true early G1 and affect lag detection at the M-to-G1 transition. The authors should list this as a limitation of their study.
• In Figure 3G, the authors report that mitotic mRNAs persist into late G1 phase. This appears to conflict with Krenning et al. 2022 (doi: 10.7554/eLife.71356), who showed that mitotic mRNAs are degraded during the M/G1 transition. This discrepancy should be accounted for - it may reflect genuine cell-type differences (fibroblasts vs. RPE-1) or could indicate that the kinetics of degradation are faster than the resolution of the early G1 bin can capture.
• The authors speculate on the discrepancy between their data and that obtained in MCF7 cells. I wonder whether the difference stems from epithelial vs. mesenchymal cell identity rather than (or in addition to) transformation status. Additionally, MCF7 cells have a notably shorter cell cycle (~18-20 h) compared to BJ-5ta fibroblasts, meaning that for a given RNA half-life, a lag would span a proportionally larger fraction of the cycle in MCF7. This kinetic argument is biologically distinct from "cancer dysregulation" and could help reframe the discussion.

Cross-comment section
I note that both Reviewers 1 and 3 praise the quality of the data put forward. I agree with them that the manuscript is technically sound, apart from some minor details that the authors will have no problem to address (for example both Reviewer 3 and I ask for a better validation of the sorted populations).
Yet, the technical quality of the data does not compensate for what I judge to be insufficient support of the manuscript's main claim. I stand my ground on the fact that this manuscript presents a major epistemological limitation. The lack of evidence for a lag between transcription activation and gene expression does not prove that such lag does not exist, as for now it only proves that the authors are unable to detect it with their experimental design. The authors could solve this problem by 1) limiting their conclusion to the transition between G1 and S phase (this would also imply changing the title), or by 2) providing a more resolved dataset.
Overall, while I recognize the hard work of the authors and the quality of the data provided, the data provided do not prove that there is no lag between transcription and RNA maturation.

Reviewer #3 (Comments to the Authors (Required)):

In this study, Gucwa & Ikegami investigate whether there is a global, cell cycle-dependent lag between active transcription and transcript maturation. They analyze the transcriptional profile and nascent transcription of FACS-sorted asynchrnously cycling population of untransformed BJ cells. They demonstrate that unlike a prevailing model (Liu 2017 PNAS) that used chemically cell-cycle arrested cancer cells, these asynchrnous cells demonstrate very little global trancription-to-maturation lag.

The major point of the paper (the lack of lag) relies on 2 components:
1) Reliable FACS sorting of all 5 (splitting G1 into early and late) phases of the cell cycle. Based on the correspondence between the live-cell imaging and FACS sorting, which seem very clean, I am reasonably convinced of these data. I would like to see a bit more of the FACS gates used, especially how possible polyploid cells are rejected.

2) High quality RNA-seq and GRO-seq anlaysis on the same samples. These datasets seem highly reproducible across the two replicates. I am also convinced by the quality of the RNAseq/Gro-seq datasets shown.

Minor points:
- While the plots showing the inter-replicate reproducibility is very visually convincing, please include R-squared values for the duplicate RNAseq and GRO-seq datasets

Reviewer 1

1) No early S GRO-seq data. The use of intronic read as substitute is acceptable but authors should clearly state in the result section that GRO-seq do not cover early S phase. Also would be helpful to explicitly highlight that GRO-seq and intronic read signal concord at phases where both are available.

We agree that these clarifications are necessary. Accordingly, we added the following sentences in the result section.
- "*Of note, we could not obtain GRO-seq data for early S phase because of insufficient numbers of sorted cells.*"
- "*Importantly, the signal dynamics of GRO-seq and intronic reads were concordant at cell-cycle phases where both datasets were available.*"

2) Both explanations for discrepancy with Liu et al data in MCF7 cells are plausible. However, the discussion would benefit from a brief comparison of gene-levels. For example, I was wondering whether genes previously reported to show lag appear among the list of concordant genes?

We thank the reviewer for this useful suggestion. Indeed, genes previously reported to exhibit transcription-to-maturation lags show highly concordant transcriptional and mature RNA dynamics in our dataset. As a gene-level example, we now discuss *CENPE*. This gene was highlighted by Liu et al. as an example of many mitosis-related genes whose GRO-seq signals peak in G1 whereas its RNA-seq signal peaks in M phase in their dataset. We have added the GRO-seq profile of *CENPE* (**Fig. 2A**) and its GRO-seq, RNA-seq, intron dynamics (**Fig. 3C**). In our dataset, both GRO-seq and RNA-seq signals peak in late-S/G2/M, and this pattern is shared by many mitosis-related genes. One possible explanation for this discrepancy is that Liu et al. did not include G2-phase cells. Their cell-cycle time point closest to G2 was noncodazol-arrested M phase, in which transcription is globally reduced. As a result, transcription peaks occurring in G2 may have been missed in their study. We now discuss this possibility using *CENPE* as an example in Discussion.

- Result section: "*For example, CENPE, which was previously reported to exhibit a transcription peak in G1 and a mature RNA peak in M phase, showed highly concordant dynamics, with RNA-seq, GRO-seq, and intron peaks all residing in late S/G2/M*."
- Discussion section: "*Another difference is that the MCF7 study did not include G2 phase cells, whereas the present study did. In the MCF7 study, many genes with RNA-seq peaks in M-phase, including the centromeric protein gene CENPE, show transcription peaks in G1. However, this may reflect exclusion of G2 phase cells, because we observed that many mitosis-related genes, including CENPE, exhibited strong transcription peaks in late-S/G2/M cells.*"

3) Global RNA levels: the authors could discuss how variations in global RNA levels across the cell cycle would impact or bias their analysis.

We agree with the reviewer that clarification is necessary. We clarified this point, as follows: "*First, our analysis normalizes data such that global transcriptional activity and total mature RNA abundance remain constant across the cell cycle. In reality, nascent transcription may be globally higher in G2 than in other phases because of genome duplication. However, even if such global changes occur, our central conclusion would remain unaffected because the abundance of mature RNA relative to that of nascent RNA was globally maintained across the analyzed phases, based on intron/exon ratio analysis (Fig. S5E).*"

4) Figure 2C: The gene counts per nascent cluster in text (e.g. cluster a: 88, b: 531) do not match the numbers visible in the figure panels (84, 480, 515, 206, 685, 801).

We thank the reviewer for identifying these inconsistencies. The gene counts in the text have been corrected to match those shown in Figure 2C.

5) Results: "early S phase did yield sufficient cell numbers for GRO-seq" I assume it should be did NOT yield.

We thank the reviewer for identifying this typo. We have corrected the error and further clarified this point in the revised manuscript.

6) Discussion: "A recent single-cell transcriptome study in HEK293T cells also reported only a minor lag" could the authors briefly compare the methodological difference with their study

We thank the reviewer for this suggestion. We have revised the text to clarify the methodological differences between the studies. The HEK293T study used single-cell RNA-seq and inferred temporal progression through the cell cycle using pseudotime analysis, whereas our study analyzed physically sorted FUCCI-defined cell-cycle populations. Despite the methodological difference, both studies reported only minor lags between nascent transcription and mature RNA dynamics.

"*Our analysis revealed strong consistency between the cell-cycle dynamics of nascent transcription and mature RNA abundance in normal human fibroblasts, using sorted cell-cycle populations (Fig. 3H). … our finding is consistent with a recent single-cell transcriptome study in HEK293T cells, which measured nascent and mature RNAs using exonic and intronic reads and reported only a minor lag along pseudotime.*"

7) I think the paper would be strengthened by a brief validation of GRO-seq signal using a housekeeping gene for example to confirm an comparable efficiency across the phases.

We thank the reviewer for this helpful suggestion. We have added a housekeeping gene (*GAPDH*) and a fibroblast-marker gene (*PDGFRA*) (**Fig. 2A**), which confirm the comparable efficiency of GRO-seq across cell-cycle phases. We also added GRO-seq profiles of dynamic genes *COL1A1* and *CENPE* in this figure for comparison.

Reviewer 2

1) Although I generally consider the manuscript solid and the data of high quality, I have one major critique that I strongly believe the authors should address before publication. The main claim of the work, as it stands, is "Lack of evidence for transcription-to-RNA maturation lags extending across cell-cycle phases in fibroblasts". I consider this statement problematic because the "lack of evidence" could simply be attributed to technical limitations.
In this regard, I note that the temporal resolution of the dataset is limited, particularly in the GRO-seq experiments. In Figure 2, the time points for GRO-seq represent only Early G1, G1, and Late S/G2/M pooled together. This lack of temporal resolution severely hinders the central conclusion of the manuscript. One could claim that transcription peak and mRNA peak fall in the same phase only because there is insufficient distinction between phases. Critically, this limitation extends to the RNA-seq data as well: late S, G2, and M are also pooled into a single fraction on the mature RNA side, meaning the authors lack resolution to detect lags within that window on either the nascent or mature side of the comparison.
It is worth noting that the only cell-cycle transition where the authors have genuinely fine-grained coverage on both the nascent and mature RNA side is around G1, and that is precisely where they detect a minor lag. Rather than merely being consistent with a resolution limitation, this observation is actively predicted by it: if lags are only detectable where temporal resolution is sufficient, the simplest interpretation is that the method is underpowered to detect them elsewhere, not that they are absent.
At the moment, the only solid conclusion is that there is a lack of evidence for transcription-to-RNA maturation lags extending across the G1/S transition. I believe the conclusions would be significantly strengthened if the authors could include more precise timing. This could be achieved in a number of orthogonal ways:
• While it might be hard to separate S and G2 experimentally, it is possible to separate M phase reasonably well (e.g. by mitotic shake-off or phospho-histone H3 staining). This would immediately test whether lags from M-phase transcription to G1 mature RNA are hidden in the current pooled bin.
• The authors could synchronize their cells with thymidine or palbociclib. Although these drugs are known to alter the cell cycle profile, they do offer better temporal resolution and could serve as an orthogonal validation of the existing dataset.

We agree that the temporal resolution of our dataset limits our ability to detect certain transcription-to-maturation lags. In particular, the heterogeneity of the sorted populations and the pooling of late S, G2, and M-phase cells could prevent detection of lags within phases and occurring between late-S/G2/M. We therefore agree that our original title and conclusions overstated what can be inferred from the data. Accordingly, we have revised the title to "Lack of evidence for transcription-to-RNA maturation lags extending across G1/G2 phase boundary in fibroblasts" and have edited the Abstract, Results, and Discussion to more precisely reflect the scope of our findings. We have also expanded the Discussion to explicitly acknowledge the limited temporal resolution and heterogeneity of the sorted populations as study limitations, as quoted below. We also agree that higher temporal resolution would provide a more rigorous assessment of transcription-to-maturation dynamics throughout the cell cycle, especially those occurring within phases. We are currently pursuing higher-resolution approaches to further investigate these dynamics.

"*Third, sorted populations were heterogenous with respect to cell-cycle position. This heterogeneity could arise from inclusion of distinct cell-cycle stages within the G0/early-G1 and late-S/G2/M populations, as well as variation within phases, such as G1 and early S. In addition, minor*

contamination from other phases may because of fluctuations in FUCCI marker expression (Fig. 1B; Fig. S1B). This heterogeneity and potential contamination could blur cell-cycle dynamics and potentially reduce sensitivity for detecting certain transcription-to-maturation lags. Nevertheless, the identification of more than 2,000 genes with mature and nascent RNA dynamics, many showing biologically relevant patterns, supports the sensitivity of our methodology."

2) It would be important to show how clean a sample the authors can isolate through cell sorting. I would like to see a PI (propidium iodide) profile of the various sorted populations to assess potential contamination from cells in different cell cycle phases.

We appreciate the reviewer's suggestion. Direct assessment of DNA content in the sorted populations was not feasible in our experimental configuration because DNA dyes such as propidium iodide exhibit substantial spectral overlap with the FUCCI4 proteins (mTurquoise2, Clover, mKO2, and mMaroon2), making accurate simultaneous classification by both approaches challenging.

Although FUCCI4-based classification may not precisely correspond to cell-cycle phase definitions based on DNA content, several observations support the validity of our sorted populations. First, live-cell imaging confirmed the expected sequential appearance of the FUCCI4 markers throughout the cell cycle. Second, RNA-seq data recapitulated the established expression dynamics of numerous cell-cycle-regulated genes. Third, we detected robust cell-cycle-dependent changes in nascent and mature RNA abundance in more than 2,000 genes. These observations indicate that the sorted populations captured biologically meaningful cell-cycle states and were not dominated by extensive cross-phase contamination that would be expected to blur such dynamics.

Nevertheless, we agree that some degree of contamination among neighboring cell-cycle populations is possible. This limitation could reduce sensitivity for detecting certain transcription-to-maturation lags. We have therefore expanded the Discussion to explicitly acknowledge this limitation, as quoted in the Comment #1 answer.

3) What happens to G0 cells with this FUCCI reporter? Is it not expressed? If so, how do the authors distinguish G0 from early G1 cells? BJ-5ta fibroblasts will have some fraction of quiescent cells, and the FUCCI4 system does not cleanly distinguish G0 from early G1, as both populations would be triple-negative for mKO2/Clover/mTurquoise2. If the early G1 bin is contaminated with G0 cells, this could blur the transcriptional signature of true early G1 and affect lag detection at the M-to-G1 transition. The authors should list this as a limitation of their study.

We thank the reviewer for this important point. To minimize the fraction of quiescent cells, we seeded cells at low density 24 hours before sorting to promote active proliferation. Nonetheless, as the reviewer notes, the triple negative population could contain G0 cells. To reflect this uncertainty, we have renamed this population "G0/early G1" throughout the manuscript. We have also added this heterogeneity as a limitation in the Discussion, as quoted in the Comment #1 answer.

4) In Figure 3G, the authors report that mitotic mRNAs persist into late G1 phase. This appears to conflict with Krenning et al. 2022 (doi: 10.7554/eLife.71356), who showed that mitotic mRNAs are degraded during the M/G1 transition. This discrepancy should be accounted for - it may reflect genuine cell-type differences (fibroblasts vs. RPE-1) or could indicate that the kinetics of degradation are faster than the resolution of the early G1 bin can capture.

We thank the reviewer for bringing this study to our attention. Upon examining the paper in detail, we found that its results are largely consistent with our observations. Specifically, Krenning et al. identified two classes of mitotic transcripts: an “immediate decrease” group (53 genes) whose mRNAs decline rapidly during the M-to-G1 transition and a larger “delayed decrease” group (167 genes) whose mRNAs decline during G1. Thus, most mitotic transcripts analyzed in that study (167 of 220 genes, 76%) exhibited delayed degradation during G1 rather than immediate decline at mitotic exit. This pattern is consistent with our observation that many mitosis-related mRNAs persist into early G1 and decline later during G1. We have added a discussion of this study to the revised manuscript.

“*This observation is consistent with a previous study showing that a majority of mitosis-related gene mRNAs are degraded during G1, not immediately after mitotic exit.*”

5) The authors speculate on the discrepancy between their data and that obtained in MCF7 cells. I wonder whether the difference stems from epithelial vs. mesenchymal cell identity rather than (or in addition to) transformation status. Additionally, MCF7 cells have a notably shorter cell cycle (~18-20 h) compared to BJ-5ta fibroblasts, meaning that for a given RNA half-life, a lag would span a proportionally larger fraction of the cycle in MCF7. This kinetic argument is biologically distinct from "cancer dysregulation" and could help reframe the discussion.

We agree that differences in cell identity and transformation status may contribute to the observed discrepancies. We also agree that differences in cell-cycle duration could influence the magnitude of transcription-to-maturation lags. We have added these possibilities to the Discussion. We note that a previous single-cell study in HEK293T cells (PMID: 39009595), which also have a short cell-cycle duration, did not report extensive lags. This suggests that cell-cycle kinetics alone may not fully explain the differences between studies.

“First, the discrepancy may relate to differences in cell-cycle progression time, which is faster in MCF7 cells than in BJ-5ta cells used in this study. For a given RNA half-life, a transcription-to-maturation lag would occupy a larger fraction of the cell cycle in rapidly cycling cells. However, this does not fully explain the absence of cross-phase lags observed in HEK293T cells, which also cycle rapidly. Other possible biological contributors include dysregulation of transcription and/or RNA turnover in certain cancer cells, cell type differences, and differences in transformation state.”

Cross-comment section

I note that both Reviewers 1 and 3 praise the quality of the data put forward. I agree with them that the manuscript is technically sound, apart from some minor details that the authors will have no problem to address (for example both Reviewer 3 and I ask for a better validation of the sorted populations).

Yet, the technical quality of the data does not compensate for what I judge to be insufficient support of the manuscript's main claim. I stand my ground on the fact that this manuscript presents a major epistemological limitation. The lack of evidence for a lag between transcription activation and gene expression does not prove that such lag does not exist, as for now it only proves that the authors are unable to detect it with their experimental design. The authors could solve this problem by 1) limiting their conclusion to the transition between G1 and S phase (this would also imply changing the title), or by 2) providing a more resolved dataset.

Overall, while I recognize the hard work of the authors and the quality of the data provided, the data provided do not prove that there is no lag between transcription and RNA maturation.

We thank the reviewer for this important conceptual clarification. We agree that the *absence of detected evidence* does not imply *absence of such lags,* but rather reflects the scope and resolution of the current experimental design. As described in our response to Comment (1), we have revised the title, Abstract, Results, and Discussion to explicitly reflect this interpretation, emphasizing that our conclusions are limited to the detection of transcription-to-maturation lags across the G1/G2 boundary in this system.

Reviewer 3:

In this study, Gucwa & Ikegami investigate whether there is a global, cell cycle-dependent lag between active transcription and transcript maturation. They analyze the transcriptional profile and nascent transcription of FACS-sorted asynchrnously cycling population of untransformed BJ cells. They demonstrate that unlike a prevailing model (Liu 2017 PNAS) that used chemically cell-cycle arrested cancer cells, these asynchrnous cells demonstrate very little global trancription-to-maturation lag.

The major point of the paper (the lack of lag) relies on 2 components:
1) Reliable FACS sorting of all 5 (splitting G1 into early and late) phases of the cell cycle. Based on the correspondence between the live-cell imaging and FACS sorting, which seem very clean, I am reasonably convinced of these data. I would like to see a bit more of the FACS gates used, especially how possible polyploid cells are rejected.

We have added a representative gating strategy in Fig. S2A, showing the selection of single cells. Doublets were excluded using FSC/SSC-based gating. Regarding polyploid cells, this population cannot be directly resolved by FSC/SSC gating alone. However, substantial contamination is unlikely, as BJ-5ta parental cells do not show a detectable polyploid population in our prior characterization (see below. Also PMID: 32208162). We have clarified this limitation in the revised manuscript, as quoted below.

[Figure removed by editorial staff per authors' request]

"*Third, sorted populations were heterogenous with respect to cell-cycle position. This heterogeneity could arise from inclusion of distinct cell-cycle stages within the G0/early-G1 and late-S/G2/M populations, as well as variation within phases, such as G1 and early S. In addition, minor contamination from other phases may because of fluctuations in FUCCI marker expression (Fig. 1B; Fig. S1B). This heterogeneity and potential contamination could blur cell-cycle dynamics and potentially reduce sensitivity for detecting certain transcription-to-maturation lags. Nevertheless, the identification of more than 2,000 genes with mature and nascent RNA dynamics, many showing biologically relevant patterns, supports the sensitivity of our methodology.*"

2) High quality RNA-seq and GRO-seq anlaysis on the same samples. These datasets seem highly reproducible across the two replicates. I am also convinced by the quality of the RNAseq/Gro-seq datasets shown.
Minor points:

- While the plots showing the inter-replicate reproducibility is very visually convincing, please include R-squared values for the duplicate RNAseq and GRO-seq datasets

We appreciate this comment. We added Pearson correlation coefficient in all correlation plots.

July 2, 2026

RE: Life Science Alliance Manuscript #LSA-2026-03670-TR

Dr. Kohta Ikegami
Cincinnati Children's Hospital Medical Center
Division of Molecular Cardiovascular Biology
240 Albert Sabin Way
Cincinnati 45229

Dear Dr. Ikegami,

Thank you for submitting your revised manuscript entitled "Lack of evidence for transcription-to-RNA maturation lags across G1/G2 phase boundary in fibroblasts". Your revised manuscript was evaluated by only one of the original reviewers due to non-availability of Reviewer 2.
As you will read below, Reviewer 1 has noted that the revised manuscript has addressed their previous concerns. We would be happy to publish your paper in Life Science Alliance pending resolution of final revisions necessary to meet our formatting guidelines.

MANUSCRIPT ORGANIZATION AND FORMATTING:

To avoid unnecessary delays in the acceptance and publication of your paper, please read the following information carefully. Full guidelines are available on our Instructions for Authors page, https://www.life-science-alliance.org/authors

Submission of a paper that does not conform to Life Science Alliance guidelines will delay the acceptance of your manuscript.

-Thank you for providing a statement of data availability. We offer a minor suggestion to clarify your statement as follows, "RNA-seq and Gro-seq data are available in Gene Expression Omnibus under accession ID GSE305617..."
-Please upload your main manuscript text as an editable doc file.
-Please upload all figure files as individual ones, including the supplementary figure files; all figure legends should only appear in the main manuscript file.
-Please remove "Consent for publication".
-Please rename "Disclosure and competing interests statement" to "Conflict of Interest."
-Please add your main, supplementary figure, and table legends to the main manuscript text after the references section
-Please add a callout for Figure S4D to your main manuscript text.
-Please add the X and Bluesky handles of your host institute/organization, as well as your own, and/or one of the authors, in our system.
-Please be sure that the authorship listing and order is correct

We encourage our authors to provide original source data, particularly uncropped/-processed electrophoretic blots and spreadsheets for the main figures of the manuscript. If you would like to add source data, we would welcome one PDF/Excel-file per figure for this information. These files will be linked as supplementary "Source Data" files.

It is Life Science Alliance policy that if requested, original data images must be made available to the editors. Failure to provide original images upon request will result in unavoidable delays in publication. Please ensure that you have access to all original data images prior to final submission.

If you are planning a press release on your work, please inform us immediately to allow informing our production team and scheduling a release date.

We welcome submissions of potential cover images for the issue of LSA in which your work would appear. If you have high quality images associated with this work, please feel free to email these, with a caption, to the journal office.

LSA encourages authors to provide a 30-60 second video where the study is briefly explained. These videos will be appear embedded with the manuscript online at Life Science Alliance, and on social media to promote the published paper and authors (for examples, see https://docs.google.com/document/d/1-UWCfbE4pGcDdcgzcmiuJl2XMBJnxKYeqRvLLrLSo8s/edit?usp=sharing). Corresponding or first-authors are welcome to submit the video. Please submit only one video per manuscript. The video can be emailed to contact@life-science-alliance.org

FINAL FILES:

To upload the final version of your manuscript, please log in to your account: https://lsa.msubmit.net/cgi-bin/main.plex
You will be guided to complete the submission of your revised manuscript and to fill in all necessary information. Please get in touch in case you do not know or remember your login name.

The following items are required for acceptance.

-- An editable version of the final text (.DOC or .DOCX) is needed for copyediting (no PDFs).

-- High-resolution figure, supplementary figure and video files uploaded as individual files: See our detailed guidelines for preparing your production-ready images, https://www.life-science-alliance.org/authors

-- Summary blurb (enter in submission system): A short text summarizing in a single sentence the study (max. 200 characters including spaces). This text is used in conjunction with the titles of papers, hence should be informative and complementary to the title. It should describe the context and significance of the findings for a general readership; it should be written in the present tense and refer to the work in the third person. Author names should not be mentioned.

The license to publish form must be signed before your manuscript can be sent to production. A link to the license to publish form will be available to the corresponding author only. Please take a moment to check your funder requirements.

Reviews, decision letters, and point-by-point responses associated with peer-review at Life Science Alliance will be published, alongside the manuscript. If you do want to opt out of having the reviewer reports and your point-by-point responses displayed, please let us know immediately.

Thank you for your attention to these final processing requirements. Please revise and format the manuscript and upload materials as soon as you are able.

Thank you for this interesting contribution to the literature. We look forward to publishing your paper in Life Science Alliance.

Sincerely,

Sarita Hebbar, PhD
Scientific Editor
Life Science Alliance
http://www.lsajournal.org

Reviewer #1 (Comments to the Authors (Required)):

All my comments were addressed satisfyingly. Thus I recommend acceptance for the manuscript.

July 8, 2026

RE: Life Science Alliance Manuscript #LSA-2026-03670-TRR

Dr. Kohta Ikegami
Cincinnati Children's Hospital Medical Center
Division of Molecular Cardiovascular Biology
240 Albert Sabin Way
Cincinnati 45229

Dear Dr. Ikegami,

Thank you for submitting your Research Article entitled "Lack of evidence for transcription-to-RNA maturation lags across G1/G2 phase boundary in fibroblasts". It is a pleasure to let you know that your manuscript is now accepted for publication in Life Science Alliance. Congratulations on this interesting work.

The final published version of your manuscript will be deposited by us to PubMed Central upon publication.

Your manuscript will now progress through copyediting and proofing. It is journal policy that authors provide original data upon request.

Your article will publish open access upon publication under a CC-BY license.

Reviews, decision letters, and point-by-point responses associated with peer-review at Life Science Alliance will be published, alongside the manuscript. If you do want to opt out of having the reviewer reports and your point-by-point responses displayed, please let us know immediately.

IMPORTANT: If you will be unreachable at any time, please provide us with the email address of an alternate author. Failure to respond to routine queries may lead to unavoidable delays in publication.

Scheduling details will be available from our production department. You will receive proofs shortly before the publication date. Only essential corrections can be made at the proof stage so if there are any minor final changes you wish to make to the manuscript, please let the journal office know now.

DISTRIBUTION OF MATERIALS:
Authors are required to distribute freely any materials used in experiments published in Life Science Alliance. Authors are encouraged to deposit materials used in their studies to the appropriate repositories for distribution to researchers.

You can contact the journal office with any questions, contact@life-science-alliance.org

Again, congratulations on a very nice paper. I hope you found the review process to be constructive and are pleased with how the manuscript was handled editorially. We look forward to future exciting submissions from your lab.

Sincerely,

Sarita Hebbar, PhD
Scientific Editor
Life Science Alliance
http://www.lsajournal.org
